# The Genome Reverse Compiler: an explorative annotation tool

**DOI:** 10.1186/1471-2105-10-35

**Published:** 2009-01-27

**Authors:** Andrew S Warren, João Carlos Setubal

**Affiliations:** 1 Virginia Bioinformatics Institute, Virginia Tech, Blacksburg, VA, USA; 2 Department of Computer Science, Virginia Tech, Blacksburg, VA, USA

## Abstract

**Background:**

As sequencing costs have decreased, whole genome sequencing has become a viable and integral part of biological laboratory research. However, the tools with which genes can be found and functionally characterized have not been readily adapted to be part of the everyday biological sciences toolkit. Most annotation pipelines remain as a service provided by large institutions or come as an unwieldy conglomerate of independent components, each requiring their own setup and maintenance.

**Results:**

To address this issue we have created the Genome Reverse Compiler, an easy-to-use, open-source, automated annotation tool. The GRC is independent of third party software installs and only requires a Linux operating system. This stands in contrast to most annotation packages, which typically require installation of relational databases, sequence similarity software, and a number of other programming language modules. We provide details on the methodology used by GRC and evaluate its performance on several groups of prokaryotes using GRC's built in comparison module.

**Conclusion:**

Traditionally, to perform whole genome annotation a user would either set up a pipeline or take advantage of an online service. With GRC the user need only provide the genome he or she wants to annotate and the function resource files to use. The result is high usability and a very minimal learning curve for the intended audience of life science researchers and bioinformaticians. We believe that the GRC fills a valuable niche in allowing users to perform explorative, whole-genome annotation.

## Background

While there has been extensive work in both automated gene finding [1-4] and functional assignment [5], there are only a few automated whole-genome annotation systems available as open source projects [6-8] and none, of which we are aware, that can be used without significant setup or manual interaction. For annotation pipelines that are commercially available [9,10] or provided as a service [11-15], it is difficult to obtain and evaluate information for the methods used. By formally addressing the integration of each component in the annotation process as part of a completely automated, open source project, it may be possible to gain a further understanding of problems facing automated genome annotation as a whole.

The Genome Reverse Compiler is open source software intended for explorative annotation of prokaryotic genomic sequences. Its name and philosophy are based on analogy with a high-level programming language compiler. In this analogy, the genome is a program in a certain low-level language that humans cannot understand. Given the sequence of any prokaryotic genome, GRC produces its corresponding "high-level program" – its annotation. GRC allows the user to annotate a target genome by simply providing annotated protein sequences, in widely accepted formats, from organisms related to the target. GRC uses a similarity search against these sequences, and sequence information from the genome itself, to find protein coding genes and determine putative function of their products. We believe an integrated, open source annotation tool such as GRC benefits the life sciences community in several ways. It opens up the realm of electronic annotation to researchers who wish to annotate sequences in-house but who lack the resources to setup an annotation pipeline. Also, submission to an online annotation service may not be realistic for those wishing to annotate a large number of sequences or for sequences that do not meet with submission restrictions. GRC can provide targeted whole genome annotation since it allows users to provide the protein sequence database to be used for annotation; such a mechanism can be especially helpful in situations where users have their own curated database of sequences in addition to publicly available sequences.

In whole genome annotation, before an organism's genes can be annotated they must be found within the genomic sequence. In its current form, the GRC focuses on finding ORFs and evaluating whether they will likely be translated into protein. In making this evaluation, one consideration is sequence composition: whether the amino acid composition of the sequence is characteristic of typical coding genes found in the target organism. Some other sources of information to consider are: whether the sequence is conserved across multiple organisms (an indicator it is subject to selective pressure), whether two open reading frames overlap with one another, and the sequence length of an ORF.

Once an ORF is determined likely to be a real gene, an annotation procedure may assign some additional information. Typically this information includes the function of the gene product. Currently there is no way to computationally determine function *ab initio*. That is, to determine the function of a gene solely based on its sequence composition without reference to a similar sequence whose function is already known.

Common practice is to assign the function of genes based on sequence similarity comparisons to a database of genes whose functions are known. In many annotation procedures, the database sequence that has the top scoring, statistically significant alignment with a target gene has its function transferred to that target gene. Because functional information is frequently electronically transplanted from one sequence to another, the degree of separation between the original source of functional information and where it is applied can be great. This may cause an inappropriate functional assignment and can lead to "error propagation", where erroneous information is repeatedly applied to various sequences through multiple electronic annotations [16]. To address this situation the GRC provides several mechanisms for controlling how functions are assigned and gives the evidence for each assignment as part of the annotation.

Traditional biological nomenclature for describing genes and their products have many subtleties, redundancies, and inconsistencies. The distinctions and assumptions necessary for interpreting this information do not promote interoperability among functional genomic databases and are difficult to account for computationally. This problem can be addressed by using a structured, precisely defined system for specifying information about a gene. One such system is the Gene Ontology [17]. The Gene Ontology, or GO as it is commonly called, is a controlled vocabulary of terms that describe the molecular function, biological process, and cellular component of a gene. GO is structured as a directed acyclic graph that creates a subsumption hierarchy through its "is_a" and other directed arcs. In this hierarchy, when one node/function is assigned to a particular sequence, all parent/ancestor nodes up to the root are implicitly assigned. Using the Gene Ontology gives an added measure of precision to assigning functions to genes. By making use of evidence codes and the GO term ID numbers, we can adapt the behavior of the annotation process to the information available for a specific sequence.

The rapid accumulation and widespread availability of genomic information for prokaryotes makes it possible to use information from previous annotations of closely related organisms to annotate a newly sequenced genome. Sequencing costs are already low enough that hundreds of new prokaryotic genomes are being sequenced every year. Moreover, efforts are underway to fill the still existing "phylogenetic gaps" in the databases of prokaryotic sequences [18]. The GRC depends on this availability to create its annotations. Using prior annotation information raises several questions that can be addressed computationally. How should the assertions made in another organism's annotation impact the assertions made for the target organism? In what context are we to believe or disbelieve indications made by previous annotations? When multiple annotations are involved, how do we resolve conflicting information? In creating an integrated annotation tool we investigate possible answers to these questions and explore novel ways for determining, *in silico*, the location and function of protein coding genes as part of an integrated process.

## Implementation

### Gene Finding

Many popular gene finding algorithms perform *ab initio *by building a sequence model based on the target genomic sequence. In creating or applying this model it is possible to overly bias results against anomalous sequences, such as viral genes or recently acquired conjugated genes. GRC incorporates a gene finding module which uses information from closely related genomes. In addition to sequence similarity information, this algorithm evaluates the information content of sequences using entropy-density profiles (EDPs) introduced by Zhu et al. [4].

To evaluate whether sequences are likely to be protein coding genes we consider sequence conservation, composition, and overlap in the genome. Conservation is determined by a sequence similarity search using FSA-BLAST [19] against a user-provided sequence database (which we call the *GRC BLAST database*). Composition is evaluated using entropy-density profiles introduced by Zhu et al. [4], and subsequently used in MED 2.0 [20] and Glimmer3 [1]. EDPs are emphasized by the GRC to discriminate between likely coding and non-coding sequences when there is low scoring or no sequence similarity information for an ORF. In their previous work Zhu et al. show the efficacy of this value by testing it on several well annotated bacterial genomes. An EDP is a feature vector, based on Shannon information theory [21], used to describe the amino acid content of a sequence. In this work, we use the EDP as an additional piece of evidence to indicate the coding potential of a sequence. In the method used by Zhu et al., each sequence is mapped to its own EDP and then compared to both a representative coding and non-coding EDP, which we will refer to as the global EDPs. Each sequence is then classified as coding or non-coding based on its distance from the global EDPs in the 20-dimensional phase space. Let *p*_*i *_be the count for each amino acid in a sequence where *i *= 1, ..., 20 represents the index of a specific amino acid. For a given sequence of length *l*, let *f*_*i *_be the frequency of the *i*th amino acid where $f_{i}=\frac{p_{i}}{l}$. The Shannon entropy for the given sequence is then defined as:

$$H=-\sum_{i=1}^{20}f_{i}\log(f_{i})$$

The entropy-density for the *i*th amino acid of a sequence is defined as:

$$S_{i}=-\frac{f_{i}\log(f_{i})}{H}$$

To compute the EDP feature vector for a given sequence we compute *S*_*i *_for *i *= 1, ..., 20.

Zhu et al. demonstrate that global EDPs representing coding and non-coding sequences for all prokaryotes can act as good centers for their respective groups in the 20-dimensional phase space and as a result can be used as initial discriminators to classify a sequence as coding or non-coding [4,20]. To perform this classification, they calculate what we will call the "entropy distance ratio" (EDR). The EDR is the ratio of the relative distances to the global EDPs. The distance for a particular sequence to the global EDPs, *D*_*c *_or *D*_*nc*_, is defined as the Euclidean distance:

$$D_{\alpha }={\left(\sum_{i=1}^{20}{\left(S_{i}-S_{i}^{\alpha }\right)}^{2}\right)}^{1/2}$$

where *α *represents "*c*" for coding or "*nc*" for non-coding.

The EDR is then defined to be:

EDR = *D*_*c*_/*D*_*nc*_

The gene finding procedure for GRC is as follows: All ORFs are generated from a linear scan of the genome. Let *M *represent this set of sequences. In order to minimize the number of unnecessary overlap evaluations, we first determine the most likely start site for each ORF. The start sites are adjusted from the original maximal coordinate to the highest scoring start site. Each start site is scored according to the average frequency at which its codon occurs and how well they fit the gene model suggested by the highest scoring compatible alignment (see below). All potential start sites are placed in a priority queue based on score.

**Figure 1:**
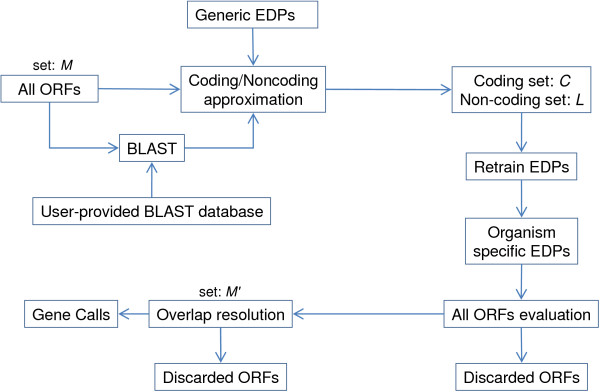
**Procedure for gene calls**. Gene calling procedure for GRC. Starting with all ORFs (set *M*), BLAST information and the generic EDPs are used to make an initial evaluation of coding and non-coding. ORFs determined to be coding go into set *C *and those ORFs that overlap them go into set *L*. The EDPs are retrained to be organism specific and are used to remove the low-scoring ORFs from *M *to create *M'*. Overlaps in *M' *are then resolved to create gene calls.

ORFs that occupy the same genomic space are said to overlap. These overlaps are evaluated and resolved by either adjusting the start coordinate of one of the offending ORFs or removing an ORF from set *M*. This process creates a set of likely coding ORFs *C *as well as a set of ORFs likely to be non-coding *L*. The likely coding and non-coding sets *C *and *L *are used to retrain the respective coding, non-coding global EDPs for the organism. Entropy distance ratios are calculated for each sequence using the new global EDPs. All ORFs with poor similarity scores and EDR scores are removed from the original set *M *creating a refined set *M'*. Using the new EDR values, a second round of overlap evaluation is performed on *M' *to determine the final set of protein coding genes (see Figure 1).

**Figure 2:**
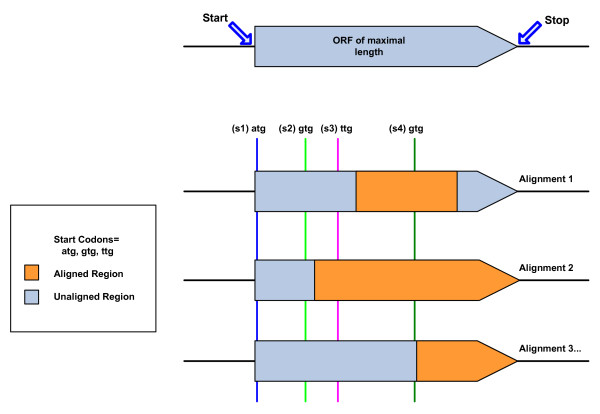
**Start site determination**. Support can come from different alignments for various start sites. Alignment 1 supports s1, s2, and s3; Alignment 2: s1 and s2; Alignment 3: s1, s2, s3, and s4. Higher start scores are given to start sites that: occur closer to a supporting alignment, occur at a higher frequency, and are supported by a higher scoring alignment.

GRC takes a heuristic approach in using information from multiple pairwise alignments and start codon frequencies to rank potential translation initiation sites. Each alignment, in the multiple pairwise alignments of the query ORF, is taken to be evidence of the start sites that occur between the aligned region and the beginning of the maximally long ORF (Figure 2). A score, *α*, is calculated for each putative start site, for each supporting alignment. As it is possible for each start site to be supported by more than one alignment, only the maximum *α *across all supporting alignments is used to represent a particular start site. We give weight to start sites that occur in closer proximity to the conserved region and that occur at a higher frequency by scoring each site according to the function *α *= *β *+ *γ *+ *δ*, where *β *is the average of the frequencies that a start site codon is found in the experimentally verified datasets of *E. coli *[22] and *B. subtilis *[23] as computed by Makita et al. [24] (ATG 0.858, GTG 0.079, TTG 0.063). These start codons and their corresponding *β *values are used by default but may be changed by the user to match the target organism. The *γ *and *δ *components are alignment specific: $\gamma =\frac{bit}{bit_{\text{self}}}$. Here *bit *represents the bit score from the alignment used to score the start and *bit*_self _is the bit score that would result from an alignment of the entire sequence to itself. This "bit fraction", *γ*, is also used in overlap evaluation and ORF removal. The last component, *δ*, is the bit score of the alignment divided by the largest bit score of all the alignments for the given ORF. This gives weight to those starts supported by longer conserved sequences.

In the "All ORFs evaluation" procedure from Figure 1, ORFs with poor similarity and EDR scores are removed from consideration as coding genes. ORFs greater than 300 bp are kept if they have an alignment *γ *score greater than 0.50 or an EDR value less than 1.0. ORFs less than 300 bp are considered small and occur at a higher frequency than those with longer sequences. Because they occur so often, small ORFs can represent a significant source of predictions and still pose a challenge for gene prediction [25]. Small ORFs are kept if they have a *γ *score greater than 0.80 or an EDR value less than one positive standard deviation from the mean EDR value of the coding set used to retrain the EDPs (Figure 1).

Genes can and do overlap in prokaryotic genomes [26,27]. Some of these overlaps are speculated to be important in regulation of gene expression [28], while others are thought to stem from phage interaction [29]. For our purposes, when evaluating overlaps present in the set of all ORFs, it is important to determine whether the overlaps represent a biological phenomenon, an error in gene coordinates, or an indication that one of the ORFs involved is not a protein coding gene. To do this, we use the BLAST hits and the EDR values for the ORFs involved to specify the amount of overlap allowed. For any given pair of overlapping ORFs it is possible for both to have alignments with significant scores. Each alignment is taken as evidence that an ORF exists as a protein coding gene (this assumes the correctness of the subject sequence and the biological significance of the alignment). If the overlap is large and there can be no reconciliation by adjusting the start site coordinate, then it is likely that one of the ORFs is not a protein coding gene and should be removed.

In a study of 198 microbial genomes Johnson et al. [30] find that 70 percent of gene overlaps are less than 15 bp and 85 percent are less than 30 bp. Extrapolating, we define a range of 1...45 bp for initial allowable overlap between ORFs. Although empirical observation indicates a maximum overlap of 45 bp, we allow up to twice that (90 bp) if the evidence for both ORFs is strong. This allows the overlap to be evaluated by the user so that he or she can make a value judgment based on the evidence. The amount of allowable overlap is defined to be:

*Allowed *= (*γ *+ (1 - *EDR*)) * 45

If the EDP for the sequence is closer to the non-coding profile, then the EDR value will be greater than one leading to a decrease in the overlap allowed. Here we use the EDR and *γ *values from the ORF that will be removed if the overlap is not resolved.

To determine which ORF in an overlapping pair will be removed we compare each ORF's alignment score, EDR, and length according to the following heuristic:

If one ORF has an alignment and the other does not, then the ORF with no alignment is removed. In all other cases the property which is determined to be the stronger discriminator is used. For two ORFs, ORF_1 _and ORF_2_, this is decided by comparing the values of the percentage difference, *D*_*S *_for each property where *S *= {*γ*, *EDR, length*}.

*D*_*S *_= |*S*_1 _- *S*_2_|/*M IN *(*S*_1_, *S*_2_)

If both ORFs have alignments, then the *D*_*S *_values for bit fraction and EDR are compared. If neither do, then the *D*_*S *_values for EDR and ORF length are compared. In both of these cases the property with the highest *D*_*S *_value is used to decide which ORF is removed.

As part of the overlap evaluation process, it may be found that altering the start coordinate of one of the conflicting ORFs will resolve an overlap. Because the highest scoring start sites are determined before overlaps are evaluated, only the alternative start sites for the low-scoring ORF of an overlapping pair are considered in resolving an overlap. Obviously if the overlap does not occur on the 5' end of an ORF, there is no point in exploring alternative start sites. Alternative start sites are considered in order of their score as given by the start site priority queue. Because the GRC stores information about multiple pairwise alignments for each ORF, it is possible that certain alignments are compatible with some start sites and not with others (Figure 2). For each start site the alignment that best fits the sequence (has the largest *α*) is used to represent the ORF.

### Functional Assignment

The functional assignments of GRC are based on associations, established through sequence similarity, between query ORFs found in the target genome and the subject sequences in the GRC BLAST database. Each association comes from an alignment that meets a user specified, e-value significance threshold. By default, the functions assigned to a query ORF are based on the annotation of one subject sequence chosen from those that have a significant alignment to the query. The subject whose alignment gives the highest *α *value (explained above), and is compatible with the start coordinate (see Figure 2), is used as the source of functional information to annotate a particular ORF (we will call this the "source subject"). If the database does not contain a sequence similar to the query sequence, then there can be no function assigned to it.

The exact information assigned to an ORF depends on the input provided to GRC. At its most basic level GRC takes a collection of amino acid FASTA files and uses it to create the BLAST database. In this case GRC simply parses the contents of the FASTA header of a subject sequence to create the annotations. If the user provides annotation tables for the corresponding sequence files then the parsing and annotation construction becomes more precise. With this level of input the product description and gene name are specified exactly and inappropriate information can be excluded. Currently the GRC supports protein annotation tables from NCBI and EMBL. The output of GRC also provides detailed information about functional assignment decisions, including confidence scores for assigned GO terms that are based on the *γ *score of the corresponding alignment.

If GO annotations are provided as additional input, GRC's functional assignment becomes more adaptable. By default GRC assigns GO terms associated with the source subject as it does in the regular annotation procedure. However, when using the Gene Ontology with GRC the user also has the option to filter the term assignments based on GO evidence codes, term depth, and GO category. Evidence codes are a three letter code associated with a Gene Ontology annotation, which specifies a source of support category for a particular annotation. Although currently the vast majority of evidence codes for prokaryotic annotations are 'IEA', *inferred by electronic annotation*, we expect this feature to be useful as the number of experimentally derived annotations and the complexity of the evidence code system increases. The user also has the option to specify which GO categories to use in making annotations (molecular function, cellular component, or biological process).

A problem encountered in transferring function is deciding which function to use when there are multiple high-scoring alignments. GRC's default practice is to transfer the function of the database sequence whose alignment best fits the ORF sequence. However, just because a subject sequence is most similar to the target gene does not guarantee that it is well annotated and is the best candidate for functional transference, *e.g. transferring a function from a 98% identical sequence experimentally determined to be glucokinase may be preferable to transferring the term "hypothetical" from a 99% identical sequence*. If the user specifies a minimum GO term depth, terms associated with the source subject, that pass the depth restriction, are assigned. If none of the GO terms from the source subject meet all the filtering criteria then GO terms are assigned from another subject that has the highest *a *score and GO terms that do meet the criteria.

GRC also has the option of generating GO "consensus annotations." Multiple, significant alignments, and their associated functions, can represent a net or distributed knowledge about the query sequence. In these cases, if only the top-scoring function is transferred, then the net knowledge is lost. We provide in GRC a feature for capturing this net knowledge by creating GO consensus annotations. Consensus annotations are intended to leverage the information distributed across the GO-DAG from multiple alignments into term assignments which have a high level of evidential support. The assumption behind consensus annotations is that multiple alignments will indicate terms that occur in relative proximity to one another within the GO-DAG and that this proximity is indicative of either a protein family with similar function or a variation in function specifics for homologous sequences in the database. The goal is to capture the proximity, and subsequent agreement, of a group of terms through these GO term assignments. Similar algorithms have been developed in GOMIT [31] and CLUGO [32] but to our knowledge no publicly available implementation of these algorithms exist.

Additionally, the user is able to specify a minimum percent coverage that the alignment must satisfy, for both the query and subject, in order to be used for function assignment. These options give a measure of control such that the annotation of an entire genome can be customized to a user's particular interests. The ability to fine-tune GO term assignment in terms of GO evidence codes, depth, and category, the use of consensus annotations, and the extensive information about functional assignment decisions contained in the output, together constitute a powerful functional assignment system not found to our knowledge in other automated annotation systems.

#### Evaluation

Also implemented in GRC is a module that allows the user to evaluate the performance of the tool with respect to a reference annotation. One part of the module provides a detailed analysis of precision and sensitivity with respect to gene finding. The details provided are meant to act as the engine to drive open-source development of the GRC and allow the user to easily evaluate the impact of his or her changes with respect to real organisms. This module also does automatic evaluation of function assignment.

Output from this module allows the review of current annotations based on evidence found in the annotation process.

### Gene Finding

Evaluating the performance of gene finding requires both a reference set of gene coordinates, *R*, and a defined system of measurement. For the purposes of using metrics, all the coordinates provided in the reference set are assumed correct. We evaluate the correctness of gene calls with respect to the starting set *M *composed of those ORFs found through a linear scan of the genome. This allows us to frame the gene finding problem for the GRC as one of classification. Given the set *M*, label each ORF in *M *as either coding (by placing it in the positive set *P*) or non-coding (by placing it in the negative set *N*). This leads to the following evaluation with respect to the reference set: every gene coordinate pair in set *P *is either a true positive (TP), a false positive (FP), or has no reference (NRP), and every coordinate pair in *N *is either a true negative (TN) or false negative (FN).

**True positive **(TP): an ORF in set *P *that is in the same frame and has the same stop site as a gene in set *R*

**False positive **(FP): an ORF in set *P *that occupies the same space as a gene in set *R *but does not meet the conditions for a TP

**No reference positive **(NRP): an ORF in set *P *that does not occupy the same space as any gene in set *R*

**False negative **(FN): an ORF in set *N *that is in the same frame and has the same stop site as a gene in set *R *(see note below)

**True negative **(TN): an ORF in set *N *that does not meet the conditions for a FN

When using the GRC, the user must specify the minimum gene length. This is the minimum nucleotide length for gene finding, which means all putative genes returned by the GRC will be greater than or equal to this number. Genes in *R *that are shorter than the minimum gene length specified are not counted as false negatives.

When measuring the performance of gene finding with respect to a reference, we wish to answer the following:

• How many of the genes in the reference set did we find (assert as being protein coding)?

• Out of the ORFs we asserted as being protein coding, how many were correct?

• And out of those correct, how many also had correct start site coordinates?

We can answer each of these questions with the following measurements:

$$\text{Sensitivity or Recall}=\frac{TP}{TP+FN}$$

$$\text{Precision or Accuracy}=\frac{TP}{TP+FP+NRP}$$

$$\text{Start Precision}=\frac{TP_{s}}{TP+FP+NRP}$$

where *TP*_*s *_= the number of true positives which have a correct start coordinate.

### Functional Assignment

In testing function assignment, we wish to measure the number of genes we assign a correct function to and, because one gene can have multiple functions, the total number of functions correctly assigned. Without a system for formal functional classification, testing function assignment can be difficult.

Comparing plain text functional descriptions will result in measuring the number of common keywords and trying to ensure that they do not convey a common biological phenomena with little meaning, *e.g. *"*protein*." To address this problem we use the Gene Ontology, which allows us to devise a more precise system for measuring function assignment performance. This system assumes that there exists a reference annotation that specifies the most specific GO terms detailing the functional characteristics of each gene in the test genome. GOA formatted files from EMBL's Integr8 project [33] and the Gene Ontology website [34] are freely available and provide this information.

Let *t *be the target gene whose functional assignment correctness we wish to determine. Let *r *be the reference gene whose function we wish to compare *t *to. There are three conditions which must be met before we can evaluate whether the function assigned to *t *is correct:

1. *t *must be a true positive in gene finding with respect to the reference gene *r*.

2. *t *must be assigned a GO term as a result of the BLAST search.

3. *r *must also be assigned a GO term from the same GO category as *t*.

**Figure 3:**
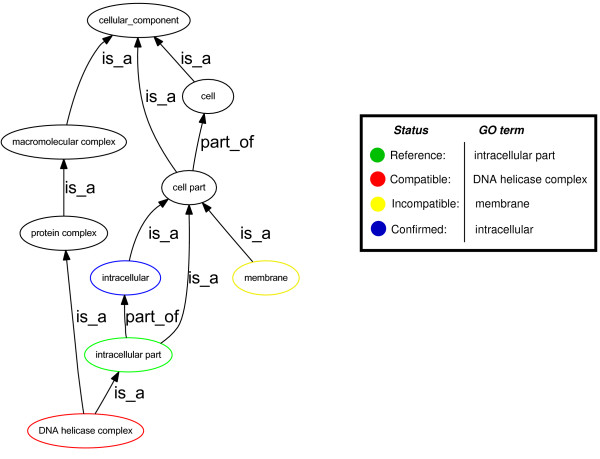
**Evaluate functional assignment using GO**. Here the term "intracellular part" represents a reference function assigned to the reference gene. The terms "intracellular", "membrane", and "DNA helicase complex" represent possible GRC GO term assignments and their evaluation with respect to the reference term.

Assuming these conditions are met, we then assign a label to each GO term that has been assigned to each TP ORF in the result set *P *(Figure 3). A term assignment is labelled **confirmed **if it coincides with or is the ancestor of a reference GO term belonging to *r*. A term is labelled **compatible **if it has as its ancestor one of the specific GO terms assigned to *r*. These represent potential refinements of the current annotation of the gene. A term is labelled **incompatible **if it does not meet the requirements to be labelled confirmed or compatible. These terms only share a common ancestor with the terms listed for *r*. Incompatible term assignments are not necessarily incorrect. The relevance of this evaluation depends on the correctness *and completeness *of the reference GO term assignments. If an evaluation of "incompatible" results from comparison to a complete (all relevant GO terms have been assigned) and correct reference annotation, then the incompatible assignment is likely incorrect. If, on the other hand, there is a relevant GO term missing in the reference annotation, then there is a chance that the GRC assigned term might be accounting for this missing information. For the purposes of GRC evaluation (see below) incompatible assignments are considered incorrect.

#### Architecture

**Figure 4:**
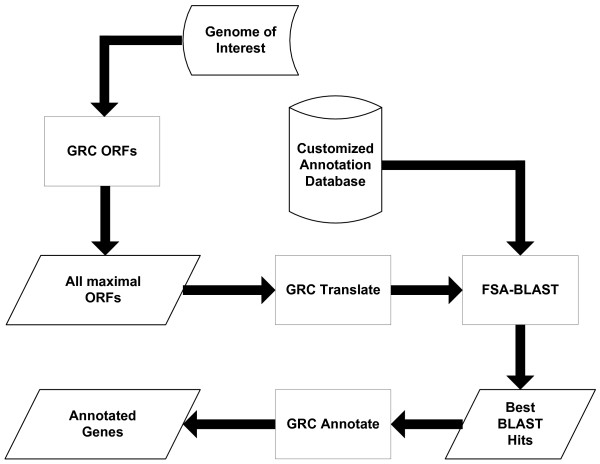
**GRC pipeline**. Internal pipeline for GRC. Maximal ORFs are found and translated. FSA-BLAST is run using the user specified database and the resulting alignments are used to call and annotate protein coding genes.

GRC is comprised of multiple components, each of which can be used independently from the annotation pipeline (Figure 4). GRC_ORFs takes a genomic sequence and finds all ORFs of maximal length. These sequences are redirected to GRC_Translate which translates the nucleic acid sequence into amino acid based on the translation table specified. Using the translated sequences as queries, FSA-BLAST performs a sequence similarity search against a user-specified database in order to identify conserved sequences and provide putative functions. GRC_Annotate takes BLAST results, and adjusts starts, assigns function, and gives putative protein coding genes as its output.

The algorithms comprising the GRC are implemented in C++ and Perl. The source code is available to download under the GNU license and comes packaged with precompiled binaries on an Intel ×86 Linux machine. Running the software requires only that the user have standard installations of g++, Perl, and Make on a Linux operating system.

We provide an additional component that can be used to easily evaluate the performance and decisions made by the GRC. GRC_Compare takes as input the output from GRC_Annotate and a reference annotation for the genome annotated. It provides an evaluation of the gene finding as well as functional assignment.

The GRC is run from the command prompt. Annotating a genome is as simple as specifying the files that contain the genomic sequence and the functionally characterized sequences from one or a number (set) of closely related organisms. We support several major formats from both NCBI and EMBL.

Example for running the GRC:

GRCv1.0.pl -g Genome.fna -d DatabaseDirectory

Because the GRC can take advantage of multiple sequence alignments in gene finding, determining start site position, and making functional assignments, the user also has the option to specify the number of top BLAST hits to use.

The output provided by the GRC increases with the amount of information provided by the user. At base level GRC provides both a list of putative protein coding genes and a list of ORFs, generated by GRC ORFs, hypothesized not to be protein coding. These lists provide the following for each ORF:

1. Highest scoring alignment values.

2. Entropy distance ratio for the sequence.

3. Assigned functions and associated confidence values.

4. Gene coordinate information.

This level of output requires only the genomic sequence and FASTA-formatted amino acid sequences for the annotation database. In this case, the functions assigned are merely the plain text descriptions obtained from FASTA headers. If the user provides additional functional information in the form of GO annotations, these will be combined with the sequence information to provide GO term assignment.

## Results and discussion

We test the performance of the GRC using leave-one-out genome annotation. For a group of related organisms, all with pre-existing annotations, each organism is annotated by the GRC using the sequences and functional descriptions from the rest of the group. Performance information is then generated using GRC_Compare to compare the GRC's annotation to that of the target organism.

In gene finding it is common practice to specify a minimum gene length [1,2,35]. Any sequences under this minimum are ignored. As the minimum gene length decreases more candidate sequences are generated from a linear scan of the genome. This increased number of sequences results in increased computation time and a higher degree of difficulty in choosing which are actually protein coding genes. Gene finding results were generated over a range of minimum gene lengths (100–300 bp) at 50 bp increments. All sequences and gene coordinate information were obtained from NCBI's RefSeq repository [36].

For each annotation the GRC was set using the following parameters:

• Number of BLAST hits to use per query = 10

• BLAST e-value threshold = .001

• Effective BLAST database size = 2879860 (char.)

• BLAST scoring matrix = BLOSUM62

To provide a frame of reference, we compare the GRC's performance to the popular gene finding program Glimmer v3.02 [1]. Glimmer was tested using the same procedure and reference files as the GRC. It should be noted for this comparison that many of the prokaryotic annotations in the Refseq repository may have been generated using Glimmer. Indeed RefSeq even provides Glimmer output files for various organisms. Glimmer was run using its iterated procedure in which it uses the sequences from the first run to create a training set for the second run. This also allows the Glimmer method to build a position weight matrix for the ribosomal binding sites and for the estimation of start-codon usage in the genome. For each run of Glimmer the default parameters were used. Only the minimum gene finding length was changed.

Glimmer parameters:

• Maximum overlap = 50

• Score threshold ≥ 30

In order to test functional assignment, we use Gene Ontology terms. The GO annotations are used as both database functions to be assigned and as reference functions. Currently, there are relatively few well curated GO annotations for multiple closely related organisms. We obtain each organism's GO annotation from EMBL's Intergr8 project [33]. These GO annotations are created through "a mixture of manual curation, and automatic inference from other annotations such as InterPro hits, UniProt keywords, and Enzyme Commission classification [33]." In testing functional assignment, we performed annotations using a minimum gene length of 300 bp.

### Test cases

We test the GRC on three groups of bacteria with varying levels of relatedness. Group 1 is composed of different strains of the species *E. coli*, Group 2 from members of the genus *Pseudomonas*, and Group 3 from members from the class *Gammaproteobacteria *(Table 1). In terms of the tree of life, both groups 1 and 2 are fairly specific and represent an availability of closely related sequences which may be lacking in some newly sequenced genomes. In all three groups we use only the primary replicon for testing the annotation capability of GRC.

**Table 1:** Test groups.

**Organism**	**Organism AC**	**ID**	**Replicon Size (Mbp)**
**Group 1 **(E. coli)			
*Escherichia coli *CFT073	NC_004431.1	CFT073	5.23
*Escherichia coli *O157 H7 EDL933	NC_009801.1	H7EDL933	5.62
*Escherichia coli *O157 H7 str. Sakai	NC_002695.1	H7Sakai	5.56
*Escherichia coli *str. K12 substr. W3110	AC_000091.1	W3110	4.71
*Escherichia coli *UTI89	NC_007946.1	UTI89	5.14
*Escherichia coli *APEC O1	NC_008563.1	APEC01	5.15

**Group 2 **(Pseudo.)			
*Pseudomonas aeruginosa *PAO1	NC_002516.2	aerPAO1	6.35
*Pseudomonas entomophila *L48	NC_008027.1	entL48	5.97
*Pseudomonas fluorescens *Pf5	NC_004129.6	fluPf5	7.17
*Pseudomonas fluorescens *PfO1	NC_007492.1	fluPfO1	6.53
*Pseudomonas syringae *pv. phaseolicola 1448A	NC_005773.3	pha1448a	6.01
*Pseudomonas putida *KT2440	NC_002947.3	puKT2440	6.27
*Pseudomonas syringae *pv. syringae B728a	NC_007005.1	syrB728a	6.18
*Pseudomonas syringae *pv. tomato str. DC3000	NC_004578.1	syrDC3000	6.49

**Group 3 **(Gamma.)			
*Escherichia coli*i str. K12 substr. W3110	AC_000091.1	W3110	4.65
*Haemophilus influenzae *Rd KW20	NC_000907.1	KW20	1.86
*Vibrio cholerae *O1 biovar eltor str. N16961	NC_002505.1	N16961	3
*Pseudomonas aeruginosa *PAO1	NC_002516.2	aerPAO1	6.35
*Coxiella burnetii *RSA 493	NC_010117.1	RSA493	2.03
*Yersinia pestis *CO92	NC_003143.1	CO92	4.72

Test groups for leave-one-out genome annotation. There is a gradation in phylogenetic distance, from closest in the E. coli group to the most distant in the Gamma group.

### Performance

**Figure 5:**
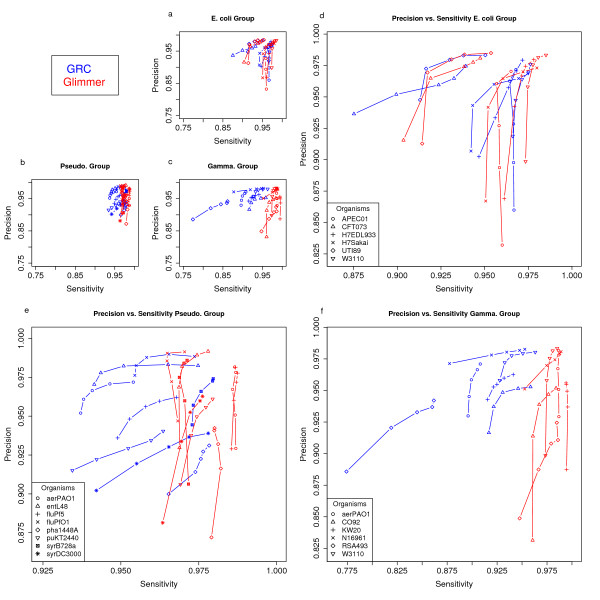
**Gene finding performance**. Performance of gene finding at increasing minimum gene length in comparison to Glimmer. Precision and Sensitivity tends to increase for each organism as the minimum gene length is increased. Lengths are 100, 150, 200, 250, and 300 bp. Panels a, b, c show the data with identical scaling. Panels d, e, f show details of Glimmer comparison for the E. coli, Pseudo, and Gamma groups. Note: The symbols for panels a, b, c match the symbols and legends of their detailed counterparts.

**Figure 6:**
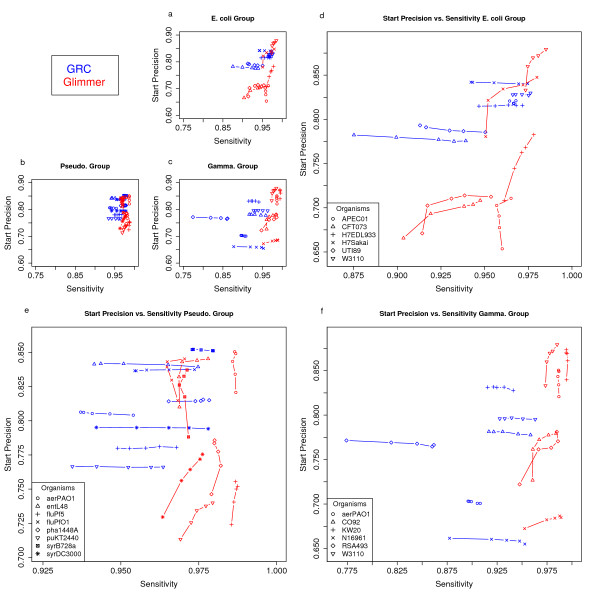
**Start site determination**. Performance of gene finding at increasing minimum gene length with respect to the fraction of TP with correct start sites (Start Precision) and Sensitivity. Panels a, b, c show the data with identical scaling. Panels d, e, f show details of Glimmer comparison for the E. coli, Pseudo, and Gamma groups. Note: The symbols for panels a, b, c match the symbols and legends of their detailed counterparts.

Using leave-one-out annotation, we test gene finding at minimum gene lengths of 100, 150, 200, 250, and 300 bases. For both the *E. coli *and *Pseudomonas *groups GRC performs well with respect to the reference files, having average values in the 0.95 – 0.99 range for both sensitivity and precision (Figure 5). Relative to Glimmer, GRC typically performs within a 2 percent margin for the sensitivity and precision measurements in the first two target groups. As seen in Figure 5, GRC tends to be more precise than Glimmer at shorter minimum lengths, but with slightly lower sensitivity. GRC is also able to consistently perform well with respect to determining the correct start site (Figure 6). GRC places heavy emphasis on sequence similarity information. As expected the precision and sensitivity with which genes are predicted, and start sites are determined, decreases as the phylogenetic distance between each member of the group increases. Although the *Gamma *group was distantly inter-related, GRC was able to achieve precision and sensitivity in the ninetieth percentile for the majority of the annotations. It is assumed that performance will continue to decrease, similar to many similarity based methods, as the relationships between the target sequences and those in the annotation database grow more distant. Great care should be taken when choosing the organisms and annotations that will make up the annotation database. Any comparison of GRC to an *ab initio *gene finder can be deemed inappropriate since GRC takes advantage of prior knowledge in making its predictions. In this case we simply wish to demonstrate the viability of a similarity based genome annotation and that GRC, despite being an explorative annotation tool, has the ability to perform satisfactory gene finding for a variety of organisms.

Regarding the results presented in Figure 5 it is important to note the following. As stated above, for the purposes of this evaluation (sensitivity measurement) we have not counted as false negatives the reference genes that are shorter than the minimum gene length. Note that the lowest threshold (100 bp) means protein sequences as short as 33 aa. Very few bona fide bacterial genes are shorter than that. In our tests, we verified that the *E. coli *group has on average 14.2 genes shorter than 100 bp, the *Pseudomonas *group has 8.2, and the Gamma group has 15.2. The precision results in turn are affected by our concept of No Reference Positives (NRPs). If the NRPs were not taken as False Positives our precision results would be better than those shown in Figure 5. As examples, using 100 bp as minimum gene length, and for the genome *E. coli *W3110 the count of NRPs is 240; for the genome *P. syringae *pv. *tomato *strain DC3000 the count of NRPs is 432 (a full account of these numbers can be found in the supplementary material: see Additional file 1).

**Figure 7:**
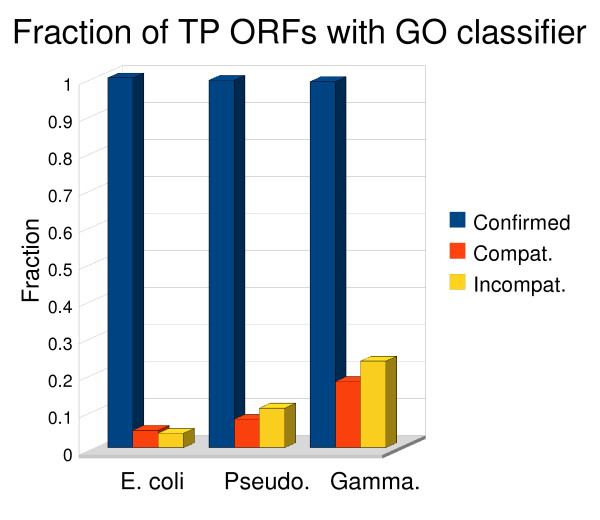
**Performance on functional assignment**. Columns show the average fraction of true positive ORFs with confirmed, compatible, and incompatible term assignments. These fractions are not additive since a TP can have a confirmed, compatible, and incompatible term assignment.

Given the functional assignment performance evaluation method we have established using GO terms, there are two ways to measure the quality of a GRC annotation. One is to compare the fraction of ORFs that have functions correctly assigned (confirmed) versus the fraction that have functions that could be incorrect (incompatible). The other is to look at the total number of terms correctly assigned. Figure 7 shows that the average fraction of ORFs with correctly assigned functions is far greater than the fraction that may have incorrect assignments. Because a single ORF can have multiple GO terms, there may be TP ORFs that are counted as having confirmed, compatible, and incompatible term assignments.

Table 2 shows the total number of confirmed, compatible, and incompatible terms for each organism for the TPs that are verifiable. Again a TP is labelled verifiable if both the GRC-ORF and its corresponding reference ORF have GO terms from the same category. This table also shows the average depth and distance for confirmed and compatible terms. The depth of a term is calculated as the minimum number of edges, over all paths, required to traverse from the root to the target term. We count the molecular function, biological process, and cellular component nodes as having depth zero. The distance is calculated as the minimum number of edges, over all paths, required to traverse from the most specific GO term in the reference to the target term.

**Table 2:** Functional prediction performance (per gene).

**Group**	**ID**	**Conf.**	**Comp.**	**Incomp.**	**Conf. Depth**	**Conf. Dist.**	**Comp. Depth**	**Comp. Dist.**
E. coli	APEC01	13278	660	485	3.77	0.09	4.9	2.49
	CFT073	13580	145	208	3.8	0.02	4.53	2.32
	H7EDL933	14731	52	66	3.83	0.01	4.65	2.29
	H7Sakai	14694	80	84	3.83	0.01	4.61	2.09
	UTI89Min	13158	118	146	3.8	0.02	4.74	2.31
	W3110Min	13855	60	67	3.84	0.01	4.58	2.6
Pseudo.	aerPAO1	12704	303	420	3.8	0.06	4.15	2.21
	entL48	10616	482	1092	3.77	0.16	4.78	2.39
	fluPf5	14101	201	371	3.8	0.02	4.02	2.03
	fluPfO1	13210	275	441	3.78	0.05	4.44	2.34
	pha1448A	11714	167	312	3.83	0.02	4.65	2.41
	puKT2440	10838	341	330	3.81	0.08	4.65	2.37
	syrB728a	12167	259	254	3.79	0.04	4.51	2.46
	syrDC3000	12002	254	253	3.8	0.05	4.59	2.43
Gamma.	aerPAO1	12631	1344	1913	3.75	0.22	4.52	2.26
	CO92	11742	770	1185	3.85	0.09	4.66	2.25
	KW20	6732	132	321	3.95	0.03	4.66	2.47
	N16961	8165	374	616	3.9	0.08	4.82	2.4
	RSA493	4036	282	335	3.9	0.1	4.77	2.58
	W3110	12913	271	302	3.84	0.03	4.81	2.54

Number of terms for each GO evaluation classifier. Only TP ORFs that have "verifiable" terms can be evaluated. Depth and distance are averaged over all term assignments for each organism.

It is possible for a confirmed or compatible annotation to be trivial in that the term assigned has no functional specificity *e.g. assign the molecular function term*. The depth and distance information in Table 2 shows that for the majority of term assignments this is not the case. As the phylogenetic relationships of an annotation group grow more distant the number of verifiable terms and confirmed terms decreases. Full tables of all GO analysis statistics can be found in the supplementary material (see Additional file 1).

Term assignments labelled incompatible do not necessarily mean the assignment is incorrect. For instance, in the annotation of *Pseudomonas *pha1448a, a protein known to be part of tryptophan synthesis (EMBL Accession = Q48QG6) was assigned the term GO:0008652 by GRC. This is a biological process term defined as "amino acid biosynthetic process." Because protein Q48QG6 was already assigned a biological process term for tryptophan metabolic process (GO:0006568) in the reference annotation, and that term was neither an ancestor nor child of the one assigned by GRC, the assignment was labelled incompatible. Also interesting to note, is that the number of compatible annotations increases as the groups become more distantly related. These annotations could be improvements on the current annotation but are also likely to include some incorrect functional assignments.

**Figure 8:**
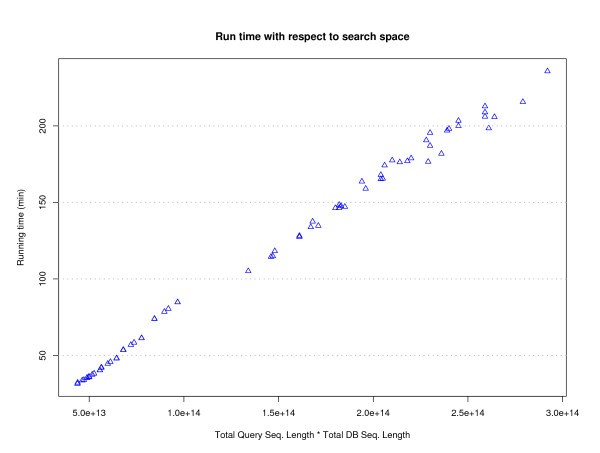
**Running time**. Total running time of GRC versus the total search space (product of total query and DB length). The main bottleneck for GRC is BLAST.

With a carefully selected annotation database the user can annotate a genome of interest in a few hours. The main bottleneck in the annotation procedure of GRC is the sequence similarity comparison. BLAST is known to scale in proportion to the product of the lengths of the query sequence and the database searched [37]. In Figure 8 we show the total run time of the GRC in relation to the product of the total query and database sequence lengths for each organism across all test groups and minimum lengths. The average total running time for all non-BLAST components of the GRC is 50 seconds with a standard deviation of 15 seconds. The total run time for non-BLAST components scales according to the amount of information available for each organism, e.g., the number of ORFs and the number of alignments. All times were obtained on a desktop computer with a 2.8 GHz processor and 1 GB of RAM.

As noted in the introduction, there exist genome annotation services available, most notably RAST [11]. Although the goals of these services and those of GRC are essentially the same (generate an automated genome annotation) the approaches adopted are quite different. Annotation services are centralized whereas GRC is a tool to be used locally and even tuned in different ways by different users. In spite of these differences we provide a comparison of the annotation results of GRC to those of RAST for *Escherichia coli *str. K12 substr. W3110 and *Pseudomonas syringae *pv. tomato str. DC3000. In Table 3 we show the performance of RAST compared to GRC for making gene calls. We also provide a comparison of the GO terms given in the RAST annotation to that of GRC (Table 4). The performance of RAST is determined by using files from EMBL's Integr8 project [33]. Although the primary form of annotation for RAST is not Gene Ontology terms, we demonstrate that the system we have established can be used to characterize the state of functional annotations on a genomic scale beyond simply listing the differences that exist. We also take the opportunity to note that the results presented above show that GRC can run in a matter of a few hours on a standard desktop computer, whereas RAST takes anywhere from one day to three days.

**Table 3:** Comparison of gene calls for RAST and GRC.

**RAST Gene Finding Comparison**			
**ID**	**Precision**	**Sensitivity**	**Start Fraction**

RAST W3110	0.98	0.98	0.80
RAST syrDC3000	0.97	0.97	0.92
GRC W3110	0.98	0.98	0.83
GRC syrDC3000	0.94	0.98	0.79

The performance of gene finding for RAST and GRC. The precision, sensitivity, and fraction of gene calls that have correct start sites for *Escherichia coli *str. K12 substr. W3110 and *Pseudomonas syringae *pv. tomato str. DC3000 (for genes > 300 bp).

**Table 4:** Comparison of GO annotations for RAST and GRC.

**RAST GO-Based Comparison**						
**ID**	**TP-Verifiable**	**Term Confirm.**	**Term Compat.**	**Term Incompat.**	**Avg.Con.Depth**	**Avg.Com.Depth**

RAST W3110	1354	1995	136	123	4.3	5.21
RAST syrDC3000	1225	1701	258	141	4.18	5.37
GRC W3110	2620	12913	271	302	3.84	4.81
GRC syrDC3000	2791	12002	254	253	3.8	4.59

The performance in making GO annotations for RAST and GRC. The number of true positives that have GO terms for the reference and the called gene are TP-verifiable. The number of GO terms found to be confirmed, compatible, and incompatible and the average depth for those terms.

## Conclusion

In GRC we have created a reliable, open-source, annotation tool which can be used for explorative annotation to investigate a genome based on the users interests. By supporting commonly available sequence and annotation formats, we provide a tool that puts very little demand on any user wishing to annotate a prokaryotic genome. GRC synthesizes information from both sequence composition and sequence similarity to minimize the deficiencies inherent in using just one. Using standards from NCBI RefSeq [36] GRC has demonstrated high precision and sensitivity in gene finding for groups of closely related prokaryotes. GRC's modular design and generic use of sequence similarity information for functional assignment and annotation means that it can be easily adapted to fit within other annotation pipelines. GRC was the automated annotation tool used in the *Pseudomonas syringae *pathovar *tomato *strain T1 genome project [38]. For this project researchers relied on the fact that GRC can use any user-provided protein sequence database; a custom database of effector protein sequences was used, and this was essential for the project's goals.

When predicted computationally, gene calls, start coordinates, and assigned functions should be taken as highly tentative until they have been curated and approved by an expert human curator. Predictions made by GRC are no different. Although GRC achieves high performance values with respect to two test groups, these groups are close phylogenetically. As the relationships between the organisms in the database and the target genome become more distant so will the applicability of annotations made by GRC. It should also be re-emphasized that the functional performance metrics were generated using reference functions (from GOA files) that were themselves electronically created. Ideally all reference information used to measure the performance of GRC should be experimentally derived. Because the GRC effectively transfers information from one organism to another, mistakes in database annotations can be propagated into a new annotation created by GRC. The confidence values, alignment information, and many of the other values output by GRC are provided so that the user can evaluate whether a gene call or functional assignment merits further investigation. These values do not provide any kind of guarantee that an *in silico *prediction will be a biological reality.

Work on GRC is ongoing. We are currently working on the following aspects. *RNA annotation*. RNA genes and features are important pieces of information in any prokaryotic genome. The fact that RNAs are usually well conserved in closely related species should make it relatively easy to include them in GRC annotations, although locating precise boundaries may be difficult. *Better use of user-provided data*. There are two main issues here. The first is the presence of experimentally derived functional assignments; those should be given preference in functional transfer, and are easily detectable in GO annotated genomes by the evidence code. The second is a user-defined special reference genome. It is often the case that among several closely related genomes there is one that is especially well annotated. For example, among *Pseudomonas syringae*, strain DC3000 is by far the best annotated. If users provide such information, GRC can be modified to make use of it and thus produce better annotations. *Metagenomics annotations*. An explorative annotation tool is in theory ideally suited for annotation of metagenomics sequences. In order for GRC to be useful in such a context a user would have to provide a BLAST database that would cover a wide range of prokaryotic species. This is not a simple task, and therefore we are planning to develop techniques that will allow the generation of a reasonably small approximation of a nonredundant and yet comprehensive set of well-annotated prokaryotic proteins.

## Availability and requirements

Project home page: 

Operating systems: Linux

Programming Languages: C++ and Perl Requirements: linux, g++, perl, Make

License: GNU General Public License. This license allows the source code to be redistributed and/or modified under the terms of the GNU General Public License as published by the Free Software Foundation. The source code for the application is available at no charge.

Any restrictions to use by non-academics: None

## Authors' contributions

AW contributed the bulk of the writing for this work, is the main programmer for the project, conceived many of the performance measurement techniques, and various other software features. JS conceived the initial GRC project idea, provided funding and guidance for this work, and has contributed to the interpretation of data and writing of the manuscript. Both AW and JS have read and approved the final manuscript.

## Supplementary Material

Additional file 1 **Supplementary data.** Full performance tables for gene finding and GO analysis for each organism. Click here for file

## References

[B1] Delcher AL, Bratke KA, Powers EC, Salzberg SL (2007). Identifying bacterial genes and endosymbiont DNA with Glimmer. Bioinformatics.

[B2] Lukashin AV, Borodovsky M (1998). GeneMark.hmm: new solutions for gene finding. Nucleic Acids Res.

[B3] Nielsen P, Krogh A (2005). Large-scale prokaryotic gene prediction and comparison to genome annotation. Bioinformatics.

[B4] Ouyang Z, Zhu H, Wang J, She ZS (2004). Multivariate entropy distance method for prokaryotic gene identification. J Bioinform Comput Biol.

[B5] Friedberg I (2006). Automated protein function prediction-the genomic challenge. Brief Bioinform.

[B6] Meyer F, Goesmann A, McHardy AC, Bartels D, Bekel T, Clausen J, Kalinowski J, Linke B, Rupp O, Giegerich R, Puhler A (2003). GenDB-an open source genome annotation system for prokaryote genomes. Nucleic Acids Res.

[B7] Berriman M, Rutherford K (2003). Viewing and annotating sequence data with Artemis. Brief Bioinform.

[B8] Manatee. http://manatee.sourceforge.net.

[B9] Overbeek R, Larsen N, Walunas T, D'Souza M, Pusch G, Eugene SelkovJ, Liolios K, Joukov V, Kaznadzey D, Anderson I, Bhattacharyya A, Burd H, Gardner W, Hanke P, Kapatral V, Mikhailova N, Vasieva O, Osterman A, Vonstein V, Fonstein M, Ivanova N, Kyrpides N (2003). The ERGOTM genome analysis and discovery system. Nucl Acids Res.

[B10] Frishman D, Albermann K, Hani J, Heumann K, Metanomski A, Zollner A, Mewes HW (2001). Functional and structural genomics using PEDANT. Bioinformatics.

[B11] Aziz R, Bartels D, Best A, DeJongh M, Disz T, Edwards R, Formsma K, Gerdes S, Glass E, Kubal M, Meyer F, Olsen G, Olson R, Osterman A, Overbeek R, McNeil L, Paarmann D, Paczian T, Parrello B, Pusch G, Reich C, Stevens R, Vassieva O, Vonstein V, Wilke A, Zagnitko O (2008). The RAST Server: Rapid Annotations using Subsystems Technology. BMC Genomics.

[B12] Van Domselaar GH, Stothard P, Shrivastava S, Cruz JA, Guo A, Dong X, Lu P, Szafron D, Greiner R, Wishart DS (2005). BASys: a web server for automated bacterial genome annotation. Nucleic Acids Res.

[B13] Bryson K, Loux V, Bossy R, Nicolas P, Chaillou S, Guchte M van de, Penaud S, Maguin E, Hoebeke M, Bessieres P, Gibrat JF (2006). AGMIAL: implementing an annotation strategy for prokaryote genomes as a distributed system. Nucl Acids Res.

[B14] Moriya Y, Itoh M, Okuda S, Yoshizawa AC, Kanehisa M (2007). KAAS: an automatic genome annotation and pathway reconstruction server. Nucleic Acids Res.

[B15] Vallenet D, Labarre L, Rouy Z, Barbe V, Bocs S, Cruveiller S, Lajus A, Pascal G, Scarpelli C, Medigue C (2006). MaGe: a microbial genome annotation system supported by synteny results. Nucl Acids Res.

[B16] Brenner SE (1999). Errors in genome annotation. Trends Genet.

[B17] Ashburner M, Ball CA, Blake JA, Botstein D, Butler H, Cherry JM, Davis AP, Dolinski K, Dwight SS, Eppig JT, Harris MA, Hill DP, Issel-Tarver L, Kasarskis A, Lewis S, Matese JC, Richardson JE, Ringwald M, Rubin GM, Sherlock G (2000). Gene ontology: tool for the unification of biology. The Gene Ontology Consortium. Nat Genet.

[B18] Joint Genome Institute A Genomic Encyclopedia of Bacteria and Archaea (GEBA). http://www.jgi.doe.gov/programs/GEBA/index.html.

[B19] Cameron M, Williams HE, Cannane A (2006). A deterministic finite automaton for faster protein hit detection in BLAST. J Comput Biol.

[B20] Zhu H, Hu GQ, Yang YF, Wang J, She ZS (2007). MED: a new non-supervised gene prediction algorithm for bacterial and archaeal genomes. BMC Bioinformatics.

[B21] Sloane NJA, Wyner AD (1993). Claude Elwood Shannon: Collected Papers.

[B22] Rudd KE (2000). EcoGene: a genome sequence database for Escherichia coli K-12. Nucl Acids Res.

[B23] Yada T, Totoki Y, Takagi T, Nakai K (2001). A Novel Bacterial Gene-Finding System with Improved Accuracy in Locating Start Codons. DNA Res.

[B24] Makita Y, de Hoon MJ, Danchin A (2007). Hon-yaku: a biology-driven Bayesian methodology for identifying translation initiation sites in prokaryotes. BMC Bioinformatics.

[B25] Skovgaard M, Jensen LJ, Brunak S, Ussery D, Krogh A (2001). On the total number of genes and their length distribution in complete microbial genomes. Trends in Genetics.

[B26] Veloso F, Riadi G, Aliaga D, Lieph R, Holmes DS (2005). Large-scale, multi-genome analysis of alternate open reading frames in bacteria and archaea. OMICS.

[B27] Behrens M, Sheikh J, Nataro JP (2002). Regulation of the overlapping pic/set locus in Shigella flexneri and enteroaggregative Escherichia coli. Infect Immun.

[B28] Krakauer DC, Plotkin JB (2002). Redundancy, antiredundancy, and the robustness of genomes. Proc Natl Acad Sci USA.

[B29] Prescott LM, Harley JP, Klein DA (2002). Microbiology.

[B30] Johnson ZI, Chisholm SW (2004). Properties of overlapping genes are conserved across microbial genomes. Genome Res.

[B31] Lee IY, Ho JM, Chen MS (2005). GOMIT: A Generic and Adaptive Annotation Algorithm Based on Gene Ontology Term Distributions. bibe.

[B32] Lee IY, Ho JM, Chen MS (2005). CLUGO: A Clustering Algorithm for Automated Functional Annotations Based on Gene Ontology. icdm.

[B33] Pruess M, Kersey P, Apweiler R (2005). The Integr8 project-a resource for genomic and proteomic data. In Silico Biol.

[B34] Gene Ontology website. http://www.geneontology.org.

[B35] Larsen TS, Krogh A (2003). EasyGene-a prokaryotic gene finder that ranks ORFs by statistical significance. BMC Bioinformatics.

[B36] K Pruitt TT, Maglott D (2003). RefSeq and LocusLink: NCBI gene-centered resources. Nucleic Acid Res.

[B37] Altschul SF, Madden TL, Schaffer AA, Zhang J, Zhang Z, Miller W, Lipman DJ (1997). Gapped BLAST and PSI-BLAST: a new generation of protein database search programs. Nucleic Acids Res.

[B38] Almeida NF, Yan S, Lindeberg M, Studholme DJ, Schneider DJ, Condon B, Liu H, Viana CJ, Warren A, Evans C, Kemen E, MacLean D, Angot A, Martin GB, Jones JD, Collmer A, Setubal JC, Vinatzer BA (2009). A Draft Genome Sequence of Pseudomonas syringae pv. tomato T1 Reveals a Type III Effector Repertoire Significantly Divergent from That of Pseudomonas syringae pv. tomato DC3000. Molecular Plant-Microbe Interactions.

