# Supplementary material for: The Genome Reverse Compiler: an explorative annotation tool
Source: BMC Bioinformatics. 2009 Jan 27;10:35. doi: 10.1186/1471-2105-10-35 (PMC2640359; doi:10.1186/1471-2105-10-35)
Supplement: Additional file 1 — Supplementary data. Full performance tables for gene finding and GO analysis for each organism. [file 1471-2105-10-35-S1.pdf]

Table 1: Annotation performance using GO verification. Number of TP that have term assignments which are Confirmed, Compatible, and Incompatible.

| **E. coli** | | | | | |
|---|---|---|---|---|---|
| **ID** | **TP** | **TP-Verifiable** | **TP Confirmed** | **TP Compatible** | **TP Incompatible** |
| APEC01 | 4144 | 2806 | 2806 | 472 | 283 |
| CFT073 | 4222 | 2915 | 2913 | 97 | 130 |
| H7EDL933 | 4524 | 3037 | 3036 | 39 | 47 |
| H7Sakai | 4516 | 3018 | 3017 | 58 | 62 |
| UTI89Min | 4113 | 2845 | 2845 | 89 | 94 |
| W3110Min | 3753 | 2804 | 2799 | 39 | 52 |
| **Pseudo.** | | | | | |
| **ID** | **TP** | **TP-Verifiable** | **TP Confirmed** | **TP Compatible** | **TP Incompatible** |
| aerPAO1 | 5035 | 3031 | 3011 | 236 | 307 |
| entL48 | 4597 | 2746 | 2721 | 359 | 707 |
| fluPf5 | 5583 | 3376 | 3354 | 169 | 289 |
| fluPfO1 | 5226 | 3132 | 3109 | 218 | 338 |
| pha1448A | 4502 | 2758 | 2738 | 125 | 212 |
| puKT2440 | 4742 | 2852 | 2828 | 284 | 256 |
| syrB728a | 4682 | 2839 | 2818 | 194 | 195 |
| syrDC3000 | 4857 | 2791 | 2776 | 206 | 189 |
| **Gamma.** | | | | | |
| **ID** | **TP** | **TP-Verifiable** | **TP Confirmed** | **TP Compatible** | **TP Incompatible** |
| aerPAO1 | 4797 | 2819 | 2771 | 873 | 993 |
| CO92 | 3378 | 2312 | 2308 | 511 | 742 |
| KW20 | 1459 | 1155 | 1152 | 88 | 220 |
| N16961 | 2251 | 1542 | 1533 | 269 | 372 |
| RSA493 | 1418 | 830 | 804 | 182 | 188 |
| W3110 | 3698 | 2620 | 2603 | 186 | 187 |

Table 2: Annotation performance using GO verification. Number of term assignments classified as Confirmed, Compatible, and Incompatible. Also the average depth of each class of term and the distance to the deepest, most specific, reference GO term.

| **E. coli** | | | | | | | |
|---|---|---|---|---|---|---|---|
| **ID** | **Term Confirm** | **Term Compat** | **Term Incompat** | **Avg. Con. Dist.** | **Avg. Con. Depth** | **Avg. Com. Dist** | **Avg. Com. Depth** |
| APEC01 | 13278 | 660 | 485 | 0.09 | 3.77 | 2.49 | 4.9 |
| CFT073 | 13580 | 145 | 208 | 0.02 | 3.8 | 2.32 | 4.53 |
| H7EDL933 | 14731 | 52 | 66 | 0.01 | 3.83 | 2.29 | 4.65 |
| H7Sakai | 14694 | 80 | 84 | 0.01 | 3.83 | 2.09 | 4.61 |
| UTI89Min | 13158 | 118 | 146 | 0.02 | 3.8 | 2.31 | 4.74 |
| W3110Min | 13855 | 60 | 67 | 0.01 | 3.84 | 2.6 | 4.58 |
| **Pseudo.** | | | | | | | |
| **ID** | **Term Confirm** | **Term Compat** | **Term Incompat** | **Avg. Con. Dist.** | **Avg. Con. Depth** | **Avg. Com. Dist** | **Avg. Com. Depth** |
| aerPAO1 | 12704 | 303 | 420 | 0.06 | 3.8 | 2.21 | 4.15 |
| entL48 | 10616 | 482 | 1092 | 0.16 | 3.77 | 2.39 | 4.78 |
| fluPf5 | 14101 | 201 | 371 | 0.02 | 3.8 | 2.03 | 4.02 |
| fluPfO1 | 13210 | 275 | 441 | 0.05 | 3.78 | 2.34 | 4.44 |
| pha1448A | 11714 | 167 | 312 | 0.02 | 3.83 | 2.41 | 4.65 |
| puKT2440 | 10838 | 341 | 330 | 0.08 | 3.81 | 2.37 | 4.65 |
| syrB728a | 12167 | 259 | 254 | 0.04 | 3.79 | 2.46 | 4.51 |
| syrDC3000 | 12002 | 254 | 253 | 0.05 | 3.8 | 2.43 | 4.59 |
| **Gamma.** | | | | | | | |
| **ID** | **Term Confirm** | **Term Compat** | **Term Incompat** | **Avg. Con. Dist.** | **Avg. Con. Depth** | **Avg. Com. Dist** | **Avg. Com. Depth** |
| aerPAO1 | 12631 | 1344 | 1913 | 0.22 | 3.75 | 2.26 | 4.52 |
| CO92 | 11742 | 770 | 1185 | 0.09 | 3.85 | 2.25 | 4.66 |
| KW20 | 6732 | 132 | 321 | 0.03 | 3.95 | 2.47 | 4.66 |
| N16961 | 8165 | 374 | 616 | 0.08 | 3.9 | 2.4 | 4.82 |
| RSA493 | 4036 | 282 | 335 | 0.1 | 3.9 | 2.58 | 4.77 |
| W3110 | 12913 | 271 | 302 | 0.03 | 3.84 | 2.54 | 4.81 |

Table 3: Performance in gene finding for GRC for the Gamma. group across all minimum lengths. Here DE, another type of False Negative, stands for "does not exist" and means that the corresponding ORF for a reference gene was not created in the initial scan of the genome. "Start Fraction" refers to the fraction of True positives which have start sites correctly predicted.

| **Gamma GRC** | | | | | | | | | | | |
|---|---|---|---|---|---|---|---|---|---|---|---|
| **Name** | **Min. Length** | **TP** | **FP** | **NRP** | **TN** | **FN** | **DE** | **Precision** | **Sensitivity** | **Start Correct** | **Start Fraction** |
| aerPAO1 | 100 | 4986 | 155 | 221 | 40677 | 579 | 0 | 0.93 | 0.90 | 3507 | 0.70 |
| aerPAO1 | 150 | 4982 | 133 | 156 | 30090 | 572 | 0 | 0.95 | 0.90 | 3503 | 0.70 |
| aerPAO1 | 200 | 4954 | 105 | 110 | 22914 | 544 | 11 | 0.96 | 0.90 | 3484 | 0.70 |
| aerPAO1 | 250 | 4889 | 99 | 71 | 17972 | 496 | 16 | 0.97 | 0.91 | 3433 | 0.70 |
| aerPAO1 | 300 | 4776 | 99 | 44 | 14683 | 451 | 32 | 0.97 | 0.91 | 3362 | 0.70 |
| CO92 | 100 | 3558 | 66 | 258 | 32917 | 321 | 2 | 0.92 | 0.92 | 2781 | 0.78 |
| CO92 | 150 | 3552 | 44 | 194 | 15622 | 300 | 2 | 0.94 | 0.92 | 2777 | 0.78 |
| CO92 | 200 | 3514 | 34 | 157 | 7458 | 243 | 19 | 0.95 | 0.93 | 2747 | 0.78 |
| CO92 | 250 | 3455 | 28 | 148 | 3639 | 177 | 20 | 0.95 | 0.95 | 2692 | 0.78 |
| CO92 | 300 | 3369 | 26 | 141 | 1907 | 118 | 28 | 0.95 | 0.96 | 2623 | 0.78 |
| KW20 | 100 | 1517 | 13 | 79 | 9551 | 140 | 0 | 0.94 | 0.92 | 1261 | 0.83 |
| KW20 | 150 | 1513 | 7 | 68 | 3782 | 128 | 1 | 0.95 | 0.92 | 1260 | 0.83 |
| KW20 | 200 | 1501 | 6 | 65 | 1455 | 122 | 0 | 0.95 | 0.92 | 1250 | 0.83 |
| KW20 | 250 | 1481 | 5 | 57 | 622 | 107 | 1 | 0.96 | 0.93 | 1233 | 0.83 |
| KW20 | 300 | 1439 | 5 | 51 | 265 | 82 | 8 | 0.96 | 0.94 | 1192 | 0.83 |
| N16961 | 100 | 2369 | 22 | 48 | 21855 | 313 | 18 | 0.97 | 0.88 | 1567 | 0.66 |
| N16961 | 150 | 2361 | 17 | 36 | 10514 | 203 | 4 | 0.98 | 0.92 | 1559 | 0.66 |
| N16961 | 200 | 2345 | 13 | 34 | 5130 | 150 | 14 | 0.98 | 0.93 | 1546 | 0.66 |
| N16961 | 250 | 2311 | 10 | 33 | 2514 | 112 | 16 | 0.98 | 0.95 | 1522 | 0.66 |
| N16961 | 300 | 2258 | 10 | 30 | 1335 | 89 | 23 | 0.98 | 0.95 | 1479 | 0.66 |
| RSA493 | 100 | 1536 | 76 | 122 | 11076 | 436 | 12 | 0.89 | 0.77 | 1185 | 0.77 |
| RSA493 | 150 | 1516 | 43 | 88 | 4657 | 332 | 3 | 0.92 | 0.82 | 1166 | 0.77 |
| RSA493 | 200 | 1498 | 33 | 75 | 1934 | 269 | 6 | 0.93 | 0.84 | 1151 | 0.77 |
| RSA493 | 250 | 1469 | 31 | 68 | 872 | 229 | 10 | 0.94 | 0.86 | 1124 | 0.76 |
| RSA493 | 300 | 1414 | 25 | 62 | 436 | 205 | 22 | 0.94 | 0.86 | 1086 | 0.77 |
| W3110 | 100 | 3911 | 48 | 123 | 35568 | 303 | 2 | 0.96 | 0.93 | 3113 | 0.80 |
| W3110 | 150 | 3905 | 35 | 77 | 19081 | 276 | 6 | 0.97 | 0.93 | 3111 | 0.80 |
| W3110 | 200 | 3872 | 27 | 62 | 10285 | 231 | 18 | 0.98 | 0.94 | 3088 | 0.80 |
| W3110 | 250 | 3790 | 26 | 54 | 5964 | 179 | 17 | 0.98 | 0.95 | 3024 | 0.80 |
| W3110 | 300 | 3687 | 25 | 49 | 3719 | 120 | 21 | 0.98 | 0.96 | 2939 | 0.80 |

Table 4: Performance in gene finding for GRC for the Pseudo. group across all minimum lengths.

| **Pseudo GRC** | | | | | | | | | | | |
|---|---|---|---|---|---|---|---|---|---|---|---|
| **Name** | **Min. Length** | **TP** | **FP** | **NRP** | **TN** | **FN** | **DE** | **Precision** | **Sensitivity** | **Start Correct** | **Start Fraction** |
| aerPAO1 | 100 | 5215 | 114 | 149 | 40790 | 349 | 1 | 0.95 | 0.94 | 4205 | 0.81 |
| aerPAO1 | 150 | 5210 | 106 | 106 | 30164 | 341 | 3 | 0.96 | 0.94 | 4200 | 0.81 |
| aerPAO1 | 200 | 5183 | 94 | 86 | 22943 | 313 | 13 | 0.97 | 0.94 | 4178 | 0.81 |
| aerPAO1 | 250 | 5112 | 92 | 62 | 17976 | 266 | 23 | 0.97 | 0.95 | 4121 | 0.81 |
| aerPAO1 | 300 | 5017 | 101 | 44 | 14666 | 211 | 31 | 0.97 | 0.95 | 4047 | 0.80 |
| entL48 | 100 | 4825 | 44 | 103 | 47099 | 300 | 0 | 0.97 | 0.94 | 4060 | 0.84 |
| entL48 | 150 | 4815 | 36 | 73 | 33030 | 279 | 9 | 0.98 | 0.94 | 4054 | 0.84 |
| entL48 | 200 | 4763 | 30 | 56 | 23564 | 228 | 18 | 0.98 | 0.95 | 4015 | 0.84 |
| entL48 | 250 | 4682 | 28 | 52 | 17396 | 145 | 24 | 0.98 | 0.97 | 3944 | 0.84 |
| entL48 | 300 | 4558 | 38 | 43 | 13469 | 91 | 27 | 0.98 | 0.97 | 3848 | 0.84 |
| fluPf5 | 100 | 5821 | 115 | 283 | 54564 | 312 | 1 | 0.94 | 0.95 | 4540 | 0.78 |
| fluPf5 | 150 | 5816 | 103 | 215 | 37444 | 285 | 3 | 0.95 | 0.95 | 4536 | 0.78 |
| fluPf5 | 200 | 5770 | 89 | 176 | 26244 | 232 | 21 | 0.96 | 0.96 | 4503 | 0.78 |
| fluPf5 | 250 | 5684 | 93 | 146 | 19227 | 184 | 38 | 0.96 | 0.96 | 4442 | 0.78 |
| fluPf5 | 300 | 5567 | 100 | 119 | 14814 | 138 | 47 | 0.96 | 0.97 | 4349 | 0.78 |
| fluPfO1 | 100 | 5472 | 46 | 87 | 51537 | 261 | 0 | 0.98 | 0.95 | 4578 | 0.84 |
| fluPfO1 | 150 | 5467 | 40 | 57 | 33715 | 256 | 2 | 0.98 | 0.95 | 4575 | 0.84 |
| fluPfO1 | 200 | 5420 | 29 | 38 | 22526 | 213 | 23 | 0.99 | 0.96 | 4539 | 0.84 |
| fluPfO1 | 250 | 5334 | 30 | 25 | 15843 | 156 | 35 | 0.99 | 0.97 | 4472 | 0.84 |
| fluPfO1 | 300 | 5205 | 36 | 25 | 11893 | 98 | 43 | 0.99 | 0.97 | 4369 | 0.84 |
| pha1448A | 100 | 4790 | 122 | 411 | 51473 | 169 | 3 | 0.90 | 0.97 | 3901 | 0.81 |
| pha1448A | 150 | 4777 | 103 | 346 | 31724 | 125 | 3 | 0.91 | 0.97 | 3891 | 0.81 |
| pha1448A | 200 | 4729 | 92 | 305 | 19892 | 103 | 15 | 0.92 | 0.98 | 3853 | 0.81 |
| pha1448A | 250 | 4637 | 86 | 278 | 13230 | 79 | 33 | 0.93 | 0.98 | 3784 | 0.82 |
| pha1448A | 300 | 4515 | 88 | 246 | 9357 | 60 | 40 | 0.93 | 0.98 | 3684 | 0.82 |
| puKT2440 | 100 | 4989 | 135 | 328 | 53125 | 348 | 2 | 0.92 | 0.93 | 3824 | 0.77 |
| puKT2440 | 150 | 4975 | 124 | 296 | 35497 | 299 | 6 | 0.92 | 0.94 | 3814 | 0.77 |
| puKT2440 | 200 | 4939 | 117 | 259 | 24027 | 226 | 21 | 0.93 | 0.95 | 3787 | 0.77 |
| puKT2440 | 250 | 4856 | 107 | 234 | 16931 | 171 | 33 | 0.93 | 0.96 | 3723 | 0.77 |
| puKT2440 | 300 | 4734 | 101 | 199 | 12580 | 126 | 53 | 0.94 | 0.96 | 3633 | 0.77 |
| syrB728a | 100 | 4950 | 84 | 207 | 51827 | 138 | 0 | 0.94 | 0.97 | 4218 | 0.85 |
| syrB728a | 150 | 4947 | 69 | 153 | 32591 | 130 | 6 | 0.96 | 0.97 | 4216 | 0.85 |
| syrB728a | 200 | 4906 | 61 | 112 | 20816 | 109 | 13 | 0.97 | 0.98 | 4181 | 0.85 |
| syrB728a | 250 | 4805 | 51 | 84 | 14100 | 78 | 23 | 0.97 | 0.98 | 4100 | 0.85 |
| syrB728a | 300 | 4667 | 53 | 71 | 10340 | 50 | 47 | 0.97 | 0.98 | 3982 | 0.85 |
| syrDC3000 | 100 | 5145 | 126 | 432 | 54620 | 311 | 5 | 0.90 | 0.94 | 4091 | 0.80 |
| syrDC3000 | 150 | 5125 | 104 | 345 | 33846 | 231 | 10 | 0.92 | 0.96 | 4075 | 0.79 |
| syrDC3000 | 200 | 5077 | 91 | 290 | 21556 | 165 | 17 | 0.93 | 0.97 | 4038 | 0.79 |
| syrDC3000 | 250 | 4975 | 85 | 252 | 14486 | 105 | 39 | 0.94 | 0.97 | 3962 | 0.79 |
| syrDC3000 | 300 | 4848 | 87 | 228 | 10312 | 73 | 36 | 0.94 | 0.98 | 3865 | 0.79 |

Table 5: Performance in gene finding for GRC for the E.coli group across all minimum lengths.

| **E. coli GRC** | | | | | | | | | | | |
|---|---|---|---|---|---|---|---|---|---|---|---|
| **Name** | **Min. Length** | **TP** | **FP** | **NRP** | **TN** | **FN** | **DE** | **Precision** | **Sensitivity** | **Start Correct** | **Start Fraction** |
| APEC01 | 100 | 4309 | 273 | 429 | 38539 | 148 | 0 | 0.86 | 0.97 | 3524 | 0.82 |
| APEC01 | 150 | 4308 | 206 | 285 | 20548 | 148 | 1 | 0.90 | 0.97 | 3524 | 0.82 |
| APEC01 | 200 | 4308 | 153 | 179 | 11043 | 145 | 4 | 0.93 | 0.97 | 3525 | 0.82 |
| APEC01 | 250 | 4296 | 95 | 72 | 6249 | 132 | 27 | 0.96 | 0.96 | 3526 | 0.82 |
| APEC01 | 300 | 4133 | 74 | 46 | 3848 | 99 | 38 | 0.97 | 0.97 | 3400 | 0.82 |
| CFT073 | 100 | 4699 | 104 | 215 | 39308 | 666 | 4 | 0.94 | 0.88 | 3676 | 0.78 |
| CFT073 | 150 | 4615 | 83 | 150 | 20829 | 493 | 23 | 0.95 | 0.90 | 3602 | 0.78 |
| CFT073 | 200 | 4502 | 69 | 121 | 11241 | 342 | 30 | 0.96 | 0.92 | 3504 | 0.78 |
| CFT073 | 250 | 4350 | 63 | 96 | 6275 | 276 | 39 | 0.96 | 0.93 | 3385 | 0.78 |
| CFT073 | 300 | 4202 | 37 | 73 | 3827 | 232 | 40 | 0.97 | 0.94 | 3271 | 0.78 |
| H7EDL933 | 100 | 4996 | 254 | 286 | 41751 | 271 | 10 | 0.90 | 0.95 | 4073 | 0.81 |
| H7EDL933 | 150 | 4957 | 199 | 155 | 22157 | 209 | 19 | 0.93 | 0.96 | 4049 | 0.82 |
| H7EDL933 | 200 | 4861 | 134 | 83 | 11908 | 157 | 27 | 0.96 | 0.96 | 3975 | 0.82 |
| H7EDL933 | 250 | 4695 | 101 | 41 | 6829 | 113 | 42 | 0.97 | 0.97 | 3846 | 0.82 |
| H7EDL933 | 300 | 4485 | 78 | 17 | 4294 | 88 | 43 | 0.98 | 0.97 | 3678 | 0.82 |
| H7Sakai | 100 | 4939 | 147 | 360 | 41561 | 299 | 4 | 0.91 | 0.94 | 4159 | 0.84 |
| H7Sakai | 150 | 4912 | 94 | 202 | 22068 | 285 | 11 | 0.94 | 0.94 | 4137 | 0.84 |
| H7Sakai | 200 | 4818 | 63 | 138 | 11906 | 208 | 17 | 0.96 | 0.96 | 4060 | 0.84 |
| H7Sakai | 250 | 4637 | 59 | 111 | 6829 | 123 | 23 | 0.96 | 0.97 | 3912 | 0.84 |
| H7Sakai | 300 | 4416 | 50 | 94 | 4293 | 87 | 28 | 0.97 | 0.97 | 3735 | 0.84 |
| UTI89 | 100 | 4599 | 92 | 162 | 38251 | 431 | 7 | 0.95 | 0.91 | 3648 | 0.79 |
| UTI89 | 150 | 4522 | 45 | 83 | 20252 | 396 | 16 | 0.97 | 0.92 | 3577 | 0.79 |
| UTI89 | 200 | 4408 | 33 | 59 | 10891 | 303 | 29 | 0.98 | 0.93 | 3473 | 0.79 |
| UTI89 | 250 | 4273 | 28 | 46 | 6138 | 247 | 36 | 0.98 | 0.94 | 3366 | 0.79 |
| UTI89 | 300 | 4100 | 28 | 42 | 3778 | 173 | 41 | 0.98 | 0.95 | 3225 | 0.79 |
| W3110 | 100 | 4073 | 120 | 240 | 35363 | 141 | 2 | 0.92 | 0.97 | 3372 | 0.83 |
| W3110 | 150 | 4052 | 85 | 140 | 18928 | 128 | 7 | 0.95 | 0.97 | 3359 | 0.83 |
| W3110 | 200 | 4003 | 63 | 87 | 10185 | 105 | 13 | 0.96 | 0.97 | 3319 | 0.83 |
| W3110 | 250 | 3887 | 55 | 66 | 5887 | 88 | 11 | 0.97 | 0.98 | 3228 | 0.83 |
| W3110 | 300 | 3737 | 41 | 51 | 3667 | 72 | 19 | 0.98 | 0.98 | 3116 | 0.83 |

Table 6: Performance in gene finding for Glimmer for the Gamma. group across all minimum lengths.

| **Gamma Glimmer** | | | | | | | | | | | |
|---|---|---|---|---|---|---|---|---|---|---|---|
| **Name** | **MinLength** | **TP** | **FP** | **NRP** | **TN** | **FN** | **DE** | **Precision** | **Sensitivity** | **Start Correct** | **Start Fraction** |
| aerPAO1 | 100 | 5492 | 237 | 181 | 0 | 0 | 73 | 0.93 | 0.99 | 4702 | 0.82 |
| aerPAO1 | 150 | 5480 | 167 | 117 | 0 | 0 | 74 | 0.95 | 0.99 | 4710 | 0.83 |
| aerPAO1 | 200 | 5431 | 119 | 65 | 0 | 0 | 78 | 0.97 | 0.99 | 4681 | 0.84 |
| aerPAO1 | 250 | 5330 | 86 | 33 | 0 | 0 | 71 | 0.98 | 0.99 | 4599 | 0.85 |
| aerPAO1 | 300 | 5188 | 76 | 21 | 0 | 0 | 71 | 0.98 | 0.99 | 4477 | 0.85 |
| CO92 | 100 | 3728 | 221 | 535 | 0 | 0 | 153 | 0.83 | 0.96 | 2870 | 0.73 |
| CO92 | 150 | 3703 | 84 | 265 | 0 | 0 | 151 | 0.91 | 0.96 | 2884 | 0.76 |
| CO92 | 200 | 3652 | 51 | 187 | 0 | 0 | 124 | 0.94 | 0.97 | 2860 | 0.77 |
| CO92 | 250 | 3566 | 35 | 165 | 0 | 0 | 86 | 0.95 | 0.98 | 2800 | 0.78 |
| CO92 | 300 | 3459 | 26 | 149 | 0 | 0 | 56 | 0.95 | 0.98 | 2716 | 0.78 |
| KW20 | 100 | 1648 | 62 | 147 | 0 | 0 | 9 | 0.89 | 0.99 | 1436 | 0.84 |
| KW20 | 150 | 1635 | 21 | 89 | 0 | 0 | 7 | 0.94 | 1 | 1426 | 0.86 |
| KW20 | 200 | 1615 | 13 | 73 | 0 | 0 | 8 | 0.95 | 1 | 1415 | 0.87 |
| KW20 | 250 | 1580 | 9 | 66 | 0 | 0 | 9 | 0.95 | 0.99 | 1388 | 0.87 |
| KW20 | 300 | 1520 | 10 | 60 | 0 | 0 | 9 | 0.96 | 0.99 | 1331 | 0.87 |
| N16961 | 100 | 2572 | 50 | 82 | 0 | 0 | 128 | 0.95 | 0.95 | 1763 | 0.67 |
| N16961 | 150 | 2503 | 18 | 60 | 0 | 0 | 65 | 0.97 | 0.97 | 1720 | 0.68 |
| N16961 | 200 | 2466 | 16 | 49 | 0 | 0 | 43 | 0.97 | 0.98 | 1698 | 0.68 |
| N16961 | 250 | 2409 | 9 | 42 | 0 | 0 | 30 | 0.98 | 0.99 | 1660 | 0.69 |
| N16961 | 300 | 2344 | 7 | 39 | 0 | 0 | 26 | 0.98 | 0.99 | 1610 | 0.68 |
| RSA493 | 100 | 1880 | 146 | 189 | 0 | 0 | 104 | 0.85 | 0.95 | 1463 | 0.72 |
| RSA493 | 150 | 1789 | 85 | 142 | 0 | 0 | 62 | 0.89 | 0.97 | 1427 | 0.76 |
| RSA493 | 200 | 1736 | 68 | 108 | 0 | 0 | 37 | 0.91 | 0.98 | 1377 | 0.76 |
| RSA493 | 250 | 1684 | 68 | 97 | 0 | 0 | 24 | 0.91 | 0.99 | 1350 | 0.77 |
| RSA493 | 300 | 1616 | 43 | 89 | 0 | 0 | 25 | 0.92 | 0.98 | 1296 | 0.78 |
| W3110 | 100 | 4104 | 169 | 294 | 0 | 0 | 112 | 0.9 | 0.97 | 3560 | 0.83 |
| W3110 | 150 | 4081 | 74 | 105 | 0 | 0 | 106 | 0.96 | 0.97 | 3573 | 0.86 |
| W3110 | 200 | 4029 | 39 | 62 | 0 | 0 | 92 | 0.98 | 0.98 | 3538 | 0.87 |
| W3110 | 250 | 3909 | 30 | 44 | 0 | 0 | 77 | 0.98 | 0.98 | 3435 | 0.87 |
| W3110 | 300 | 3771 | 22 | 42 | 0 | 0 | 57 | 0.98 | 0.99 | 3335 | 0.88 |

Table 7: Performance in gene finding for Glimmer for the Pseudo. group across all minimum lengths.

| Pseudo Glimmer | | | | | | | | | | | |
|---|---|---|---|---|---|---|---|---|---|---|---|
| **Name** | **MinLength** | **TP** | **FP** | **NRP** | **TN** | **FN** | **DE** | **Precision** | **Sensitivity** | **Start Correct** | **Start Fraction** |
| aerPAO1 | 100 | 5492 | 237 | 181 | 0 | 0 | 73 | 0.93 | 0.99 | 4702 | 0.82 |
| aerPAO1 | 150 | 5480 | 167 | 117 | 0 | 0 | 74 | 0.95 | 0.99 | 4710 | 0.83 |
| aerPAO1 | 200 | 5431 | 119 | 65 | 0 | 0 | 78 | 0.97 | 0.99 | 4681 | 0.84 |
| aerPAO1 | 250 | 5330 | 86 | 33 | 0 | 0 | 71 | 0.98 | 0.99 | 4599 | 0.85 |
| aerPAO1 | 300 | 5188 | 76 | 21 | 0 | 0 | 71 | 0.98 | 0.99 | 4477 | 0.85 |
| entL48 | 100 | 4967 | 202 | 174 | 0 | 0 | 160 | 0.93 | 0.97 | 4187 | 0.81 |
| entL48 | 150 | 4943 | 90 | 70 | 0 | 0 | 160 | 0.97 | 0.97 | 4188 | 0.83 |
| entL48 | 200 | 4857 | 51 | 38 | 0 | 0 | 152 | 0.98 | 0.97 | 4138 | 0.84 |
| entL48 | 250 | 4727 | 29 | 22 | 0 | 0 | 124 | 0.99 | 0.97 | 4015 | 0.84 |
| entL48 | 300 | 4573 | 25 | 13 | 0 | 0 | 103 | 0.99 | 0.98 | 3887 | 0.85 |
| fluPf5 | 100 | 6045 | 264 | 198 | 0 | 0 | 89 | 0.93 | 0.99 | 4568 | 0.72 |
| fluPf5 | 150 | 6020 | 154 | 95 | 0 | 0 | 84 | 0.96 | 0.99 | 4572 | 0.74 |
| fluPf5 | 200 | 5945 | 100 | 72 | 0 | 0 | 78 | 0.97 | 0.99 | 4534 | 0.75 |
| fluPf5 | 250 | 5832 | 81 | 52 | 0 | 0 | 74 | 0.98 | 0.99 | 4447 | 0.75 |
| fluPf5 | 300 | 5676 | 69 | 39 | 0 | 0 | 76 | 0.98 | 0.99 | 4340 | 0.76 |
| fluPfO1 | 100 | 5552 | 179 | 132 | 0 | 0 | 182 | 0.95 | 0.97 | 4670 | 0.81 |
| fluPfO1 | 150 | 5532 | 101 | 57 | 0 | 0 | 193 | 0.97 | 0.97 | 4674 | 0.83 |
| fluPfO1 | 200 | 5457 | 60 | 20 | 0 | 0 | 199 | 0.99 | 0.96 | 4631 | 0.84 |
| fluPfO1 | 250 | 5331 | 46 | 5 | 0 | 0 | 194 | 0.99 | 0.96 | 4534 | 0.84 |
| fluPfO1 | 300 | 5188 | 41 | 4 | 0 | 0 | 158 | 0.99 | 0.97 | 4420 | 0.85 |
| pha1448A | 100 | 4864 | 224 | 490 | 0 | 0 | 104 | 0.87 | 0.98 | 3797 | 0.75 |
| pha1448A | 150 | 4817 | 117 | 323 | 0 | 0 | 88 | 0.92 | 0.98 | 3785 | 0.77 |
| pha1448A | 200 | 4756 | 83 | 264 | 0 | 0 | 91 | 0.93 | 0.98 | 3762 | 0.78 |
| pha1448A | 250 | 4654 | 67 | 226 | 0 | 0 | 95 | 0.94 | 0.98 | 3699 | 0.78 |
| pha1448A | 300 | 4523 | 69 | 208 | 0 | 0 | 92 | 0.94 | 0.98 | 3608 | 0.79 |
| puKT2440 | 100 | 5179 | 227 | 310 | 0 | 0 | 165 | 0.91 | 0.97 | 3856 | 0.71 |
| puKT2440 | 150 | 5135 | 150 | 191 | 0 | 0 | 145 | 0.94 | 0.97 | 3834 | 0.73 |
| puKT2440 | 200 | 5053 | 113 | 155 | 0 | 0 | 133 | 0.95 | 0.97 | 3793 | 0.73 |
| puKT2440 | 250 | 4945 | 94 | 135 | 0 | 0 | 115 | 0.96 | 0.98 | 3717 | 0.74 |
| puKT2440 | 300 | 4812 | 83 | 112 | 0 | 0 | 101 | 0.96 | 0.98 | 3622 | 0.74 |
| syrB728a | 100 | 4944 | 226 | 285 | 0 | 0 | 144 | 0.91 | 0.97 | 4075 | 0.79 |
| syrB728a | 150 | 4933 | 98 | 108 | 0 | 0 | 150 | 0.96 | 0.97 | 4113 | 0.82 |
| syrB728a | 200 | 4871 | 59 | 66 | 0 | 0 | 157 | 0.97 | 0.97 | 4073 | 0.83 |
| syrB728a | 250 | 4760 | 37 | 40 | 0 | 0 | 146 | 0.98 | 0.97 | 3994 | 0.83 |
| syrB728a | 300 | 4627 | 34 | 32 | 0 | 0 | 137 | 0.99 | 0.97 | 3902 | 0.84 |
| syrDC3000 | 100 | 5269 | 281 | 428 | 0 | 0 | 200 | 0.88 | 0.96 | 4049 | 0.73 |
| syrDC3000 | 150 | 5202 | 129 | 240 | 0 | 0 | 164 | 0.93 | 0.97 | 4031 | 0.76 |
| syrDC3000 | 200 | 5113 | 85 | 170 | 0 | 0 | 146 | 0.95 | 0.97 | 3973 | 0.76 |
| syrDC3000 | 250 | 4993 | 66 | 143 | 0 | 0 | 126 | 0.96 | 0.98 | 3905 | 0.77 |
| syrDC3000 | 300 | 4839 | 65 | 122 | 0 | 0 | 118 | 0.96 | 0.98 | 3803 | 0.78 |

Table 8: Performance in gene finding for Glimmer for the E.coli group across all minimum lengths.

| **E.coli Glimmer** | | | | | | | | | | | |
|---|---|---|---|---|---|---|---|---|---|---|---|
| **Name** | **MinLength** | **TP** | **FP** | **NRP** | **TN** | **FN** | **DE** | **Precision** | **Sensitivity** | **Start Correct** | **Start Fraction** |
| APEC01 | 100 | 4279 | 351 | 513 | 0 | 0 | 178 | 0.83 | 0.96 | 3026 | 0.65 |
| APEC01 | 150 | 4272 | 231 | 277 | 0 | 0 | 185 | 0.89 | 0.96 | 3051 | 0.68 |
| APEC01 | 200 | 4271 | 171 | 165 | 0 | 0 | 186 | 0.93 | 0.96 | 3068 | 0.69 |
| APEC01 | 250 | 4263 | 118 | 57 | 0 | 0 | 192 | 0.96 | 0.96 | 3078 | 0.7 |
| APEC01 | 300 | 4121 | 89 | 37 | 0 | 0 | 149 | 0.97 | 0.97 | 2992 | 0.71 |
| CFT073 | 100 | 4853 | 190 | 258 | 0 | 0 | 518 | 0.92 | 0.9 | 3357 | 0.67 |
| CFT073 | 150 | 4716 | 72 | 100 | 0 | 0 | 415 | 0.96 | 0.92 | 3319 | 0.69 |
| CFT073 | 200 | 4573 | 49 | 73 | 0 | 0 | 301 | 0.97 | 0.94 | 3243 | 0.7 |
| CFT073 | 250 | 4401 | 40 | 61 | 0 | 0 | 264 | 0.98 | 0.94 | 3125 | 0.7 |
| CFT073 | 300 | 4239 | 28 | 55 | 0 | 0 | 235 | 0.98 | 0.95 | 3022 | 0.71 |
| H7EDL933 | 100 | 5079 | 383 | 383 | 0 | 0 | 205 | 0.87 | 0.96 | 3869 | 0.71 |
| H7EDL933 | 150 | 5014 | 196 | 110 | 0 | 0 | 171 | 0.94 | 0.97 | 3879 | 0.74 |
| H7EDL933 | 200 | 4900 | 124 | 45 | 0 | 0 | 145 | 0.97 | 0.97 | 3830 | 0.76 |
| H7EDL933 | 250 | 4721 | 103 | 21 | 0 | 0 | 129 | 0.97 | 0.97 | 3706 | 0.77 |
| H7EDL933 | 300 | 4515 | 81 | 13 | 0 | 0 | 101 | 0.98 | 0.98 | 3597 | 0.78 |
| H7Sakai | 100 | 4985 | 292 | 472 | 0 | 0 | 259 | 0.87 | 0.95 | 4119 | 0.78 |
| H7Sakai | 150 | 4958 | 113 | 194 | 0 | 0 | 250 | 0.94 | 0.95 | 4166 | 0.82 |
| H7Sakai | 200 | 4844 | 58 | 120 | 0 | 0 | 199 | 0.96 | 0.96 | 4090 | 0.83 |
| H7Sakai | 250 | 4649 | 46 | 98 | 0 | 0 | 134 | 0.97 | 0.97 | 3939 | 0.84 |
| H7Sakai | 300 | 4440 | 39 | 84 | 0 | 0 | 91 | 0.97 | 0.98 | 3796 | 0.85 |
| UTI89 | 100 | 4607 | 203 | 237 | 0 | 0 | 432 | 0.91 | 0.91 | 3228 | 0.67 |
| UTI89 | 150 | 4526 | 65 | 78 | 0 | 0 | 408 | 0.97 | 0.92 | 3225 | 0.7 |
| UTI89 | 200 | 4413 | 41 | 50 | 0 | 0 | 327 | 0.98 | 0.93 | 3164 | 0.71 |
| UTI89 | 250 | 4276 | 30 | 41 | 0 | 0 | 280 | 0.98 | 0.94 | 3074 | 0.71 |
| UTI89 | 300 | 4114 | 28 | 35 | 0 | 0 | 200 | 0.98 | 0.95 | 2951 | 0.71 |
| W3110 | 100 | 4104 | 169 | 294 | 0 | 0 | 112 | 0.9 | 0.97 | 3560 | 0.83 |
| W3110 | 150 | 4081 | 74 | 105 | 0 | 0 | 106 | 0.96 | 0.97 | 3573 | 0.86 |
| W3110 | 200 | 4029 | 39 | 62 | 0 | 0 | 92 | 0.98 | 0.98 | 3538 | 0.87 |
| W3110 | 250 | 3909 | 30 | 44 | 0 | 0 | 77 | 0.98 | 0.98 | 3435 | 0.87 |
| W3110 | 300 | 3771 | 22 | 42 | 0 | 0 | 57 | 0.98 | 0.99 | 3335 | 0.88 |
